# Biopsychosocial Consideration of *Ikigai* in Older Adults in Japan through a Cross-Sectional Study

**DOI:** 10.3390/geriatrics9030078

**Published:** 2024-06-08

**Authors:** Takaharu Goto, Shinji Fujiwara, Tomoya Koda, Takashi Matsuda, Mio Kitamura, Yasuhiko Shirayama, Tetsuo Ichikawa

**Affiliations:** 1 Department of Prosthodontics & Oral Rehabilitation, Tokushima University Graduate School of Biomedical Sciences, Tokushima 770-8504, Japan; tak510@tokushima-u.ac.jp (T.G.); matsuda.takashi.1@tokushima-u.ac.jp (T.M.); 2 Mima Municipal Koyadaira Clinic, Tokushima 777-0302, Japan; syfujiwara@jichi.ac.jp; 3 Kamikatsu Town Clinic, Tokushima 771-4505, Japan; m04026tk@gmail.com; 4 Department of Community Medical and Welfare, Tokushima University Graduate School of Biomedical Sciences, Tokushima 770-8504, Japan; kitamura.mio@tokushima-u.ac.jp (M.K.); shirayama@tokushima-u.ac.jp (Y.S.)

**Keywords:** *Ikigai*, affective psychological conditions, biopsychosocial model, well-being, quality of life

## Abstract

*Ikigai*—a Japanese concept that includes elements such as life’s purpose and meaning—has been reported to be associated with various systemic health conditions, such as the risk of developing physical dysfunction or death in older adults. However, there are no reports that comprehensively examine the psychological and social aspects of *Ikigai*. We attempted to clarify the characteristics of *Ikigai* by examining it from a biopsychosocial model using physical, psychological, and social perspectives through a cross-sectional study on sarcopenia, frailty and healthy life expectancy in a hilly and mountainous area of Japan. Koyadaira in Mima City, which is located in a hilly and mountainous region on Shikoku Island in Japan, was targeted. This cross-sectional study included 105 outpatients aged 65 and over, with an average age of 79.02 ± 6.91 years. *Ikigai* (self-rating score on a scale of 0 (no *Ikigai*) to 5 (the highest *Ikigai*)) participants’ level of physical activity (the Physical Activity Scale for the Elderly, PASE), degree of depression (the Geriatric Depression-15 Scale, GDS-15), cognitive function (the Mini-Mental State Examination, MMSE) and social isolation (the abbreviated Lubben Social Network Scale, LSNS-6) was assessed. Significant positive correlations were found between PASE and MMSE. The LSNS-6 significantly correlated with the MMSE and GDS-15. In a path model, out of four paths from PASE, GDS-15, MMSE, and LSNS-6 to *Ikigai*, the path from the GDS-15 alone was significant (correlation coefficient −0.271, *p* < 0.01). The adaptability of this model was good. This study indicates that depressive status has a large impact on *Ikigai*, along with physical, cognitive, and social conditions; thus, it is appropriate to consider that an affective psychological status, such as depressive symptoms, is a fundamental condition for having *Ikigai*.

## 1. Introduction

The global older adult population has increased in the recent past. According to the World Population Prospects report (2019) [1], the aging rate, which was around 9.1% in 2019, is expected to reach 11.7% in 2030 and 15.9% in 2050. Population aging is a crucial issue in most developed countries. Many countries have been working on extending healthy life expectancy through various measures ever since the World Health Organization (WHO) proposed the index of healthy life expectancy in 2000 [2]. In particular, Japan’s aging rate was 29.1% as of 2022, the world’s highest, after surpassing Italy in 2005, and Japan’s policies to extend healthy life expectancy and countermeasures for older adults are attracting attention.

Psychological factors play an important role in morbidity risk and mortality, and are also an important factor for comprehending the quality of life (QoL) and well-being of the older adults [3,4,5]. One psychological factor is *Ikigai*, a concept that includes elements such as life’s purpose and meaning. It has been used in Japan to describe that which gives an individual value in one’s life and makes it worthwhile, and the concept has been widely quoted all over the world [6,7,8,9]. The Oxford Encyclopedia [10] defines it as “a motivating force; something or someone that gives a person a sense of purpose or a reason for living”. More recently, García and Miralles defined *Ikigai* as an overlap of four factors: what you love (passion), what you are good at (profession), what you can be paid for (vocation), and what the world needs (mission) [11].

However, the original Japanese connotation is not necessarily as described above, and the four factors are not always equally important. From the Japanese viewpoint, Naoi states that *Ikigai* is divided into its target and mental state for the target, and two factors, foundational state and performance goal, are needed to have *Ikigai* [12]. Additionally, Namihira reported that *Ikigai* must be considered based on the three concepts: to share *Ikigai,* to have a more concrete target of *Ikigai,* and to be significant through the ages [13].

Having *Ikigai* has been reported to be associated with various systemic health conditions in older adults. A 7-year prospective cohort study by Sone et al. reported that the risk of death in those who did not have *Ikigai* increased compared to those who had *Ikigai* [14]. Mori et al. reported that having *Ikigai* significantly reduces the risk of developing physical dysfunction in the future [15]. However, there are no reports that comprehensively examine the psychological and social aspects of *Ikigai* in addition to its physical aspects. The WHO Charter defines health as “a state of complete physical, mental, spiritual, and social well-being, not merely the absence of disease or infirmity” [16]. Engel also presents the biopsychosocial model, in which health is understood and addressed within biopsychosocial systems [17]. In the medical field, there is a shift from the conventional reductionistic biomedical model to the biopsychosocial model in understanding and treating clinical conditions. In other words, patients are now being evaluated holistically, with an emphasis on the relationship between biological, psychological, and social factors and the degree of their influence on the patient’s condition. This approach is extremely useful for evaluating *Ikigai*, which is thought to be based on the interconnectedness of physical, psychological, and social factors, and leads to a comprehensive assessment of *Ikigai*.

Based on the aforementioned Japanese way of perceiving *Ikigai*, it is hypothesized that the *Ikigai* of older adults is affected by the perspective of a holistic individual, that is, their physical, psychological, and social conditions. Focusing on older adults living in hilly and mountainous regions with a particularly high aging rate in Japan, we comprehensively examined the physical, psychological, including affective and cognitive aspects, and social aspects of *Ikigai*, and attempted to clarify the biopsychosocial characteristics of *Ikigai* in a cross-sectional study.

## 2. Materials and Methods

### 2.1. Study Area and Participants

An ongoing cohort study is currently investigating the relationship between healthy life expectancy and oral, cognitive, and physical functions; social factors such as participation in community activities; and nutritional intake among older adults in hilly and mountainous regions in Japan, titled “A Population-Based Prospective Study on the Roles of Shopping, Oral Function, Nutrition, and Genetics in Sarcopenia, Frailty and Healthy Life Expectancy in Mima City” (The Mima-SONGS Study). This study commenced in 2018 with the aim of developing comprehensive efforts to extend the healthy life expectancy of older adults. The target area was Koyadaira in Mima City, located in the western part of Tokushima Prefecture (on Shikoku Island to the west of Osaka) in Japan, and approximately 95% of this area is mountainous. In 2018, Koyadaira’s population was 645 and its aging rate was 60%; thus, it is said that Koyadaira is highly likely to disappear in the near future. This cross-sectional study included 105 outpatients aged 65 and over, with an average age of 79.02 ± 6.91 years, who regularly visit the Mima Municipal Koyadaira Clinic, the medical institution in the area. All participants provided written informed consent prior to study participation. This study was approved by the Ethics Committee of Kyoto Medical Center (approval number: 17-032). It was conducted according to the criteria set out by the Declaration of Helsinki. Participants gave written informed consent before taking part in the study.

### 2.2. Measurements

*Ikigai* was assessed through the participants’ response to the question, “Do you have any pleasure or *Ikigai*?” on the basis of previous report [18]. As an operational definition, this subjective score of 0 indicated having no *Ikigai* and 5 indicated having higher *Ikigai*. The participants were asked to rate self-rated *Ikigai* on this scale of 0 to 5, with higher scores representing higher *Ikigai*.

Regarding the physical aspect, the participants’ level of physical activity was assessed using the Japanese version of the Physical Activity Scale for the Elderly (PASE). The PASE, originally developed by Washburn et al. [19], has been internationally and widely used to assess physical activity in older adults [20,21]. The PASE score includes information on leisure, household, and occupational activities, and is calculated by the amount of time spent (hours/week) on or participation (yes/no) in the said activity over a 1-week period. The reliability of the Japanese version of the PASE was confirmed by Hagiwara et al. [22], with an intraclass correlation coefficient of 0.65. In this study, the total physical activity score, ranging from 0 to 360 points and including scores for leisure, household, and occupational activities, was calculated as the total PASE score.

For the affective psychological assessment, the degree of depression was evaluated using the Japanese short version of the Geriatric Depression-15 Scale (GDS-15) for older adults, developed by Sheikh and Yesavage [23]. The GDS, originally developed by Yesavage et al. [24], contains 30 items and is one of the most widespread and reliable screening scales to evaluate depression among older adults [25,26]. Each item is scored dichotomously (yes/no). The total score ranges from 0 to 15 points, with higher scores representing more depressive symptoms. The reliability of the Japanese version of the GDS-15 was confirmed by Sugishita et al. [27], with a Cronbach’s alpha coefficient of 0.83.

For the cognitive psychological assessment, cognitive function was assessed using the Japanese version of the Mini-Mental State Examination (MMSE). The original version of the MMSE is the best-known and most commonly used scale for cognitive function evaluation [28]. The reliability of the Japanese version of the MMSE was confirmed by Sugishita et al. [29], with a test–retest reliability of 0.80. The total score ranges from 0 to 30 points, with higher scores indicating better cognitive functioning.

For social assessment, social isolation was assessed using the Japanese version of the abbreviated Lubben Social Network Scale (LSNS-6), reported by Lubben [30]. The LSNS-6 has been used worldwide as a tool to screen for social isolation in older adults [31], and the reliability of the Japanese version of the LSNS-6 was confirmed by Kurimoto et al. [32], with a Cronbach’s alpha coefficient of 0.82. In the LSNS-6, the total score ranges from 0 to 30 points, with a low score indicating weaker social networks and social isolation. Taking into consideration the characteristics of older people, the examiners read the questions aloud using a printed questionnaire form and evaluated if the subjects could visually confirm the answers on it. The examiners then filled in the responses from the subjects on the form. Additionally, each participant’s age and gender were recorded as demographic attributes.

### 2.3. Statistical Analysis

A one-way analysis of variance with Bonferroni post hoc tests was used for the PASE, MMSE, GDS-15, and LSNS-6 for a comparison in each *Ikigai* group. Spearman’s correlation was used to examine the relationship between PASE, MMSE, GDS-15, and LSNS-6. 

Multiple regression analysis with the *Ikigai* score as the dependent variable was conducted to examine the age and gender adjusted effects of PASE, MMSE, GDS-15, and LSNS-6 on *Ikigai*.

A path model analysis was then conducted to examine a hypothesis model of the effect of physical, psychological, and social factors on *Ikigai* based on the obtained results using a covariance structure analysis. *p* value, goodness-of-fit index (GFI), adjusted goodness-of-fit index (AGFI), comparative fit index (CFI), and root mean square error of approximation (RMSEA) were calculated to examine the model’s adaptability. A *p* value of >0.05, and scores of >0.95, >0.90, >0.97, and <0.08 for GFI, AGFI, CFI, and RMSEA, respectively, were considered to indicate a good model fit [33].

The statistical power of the regression-based path analysis was calculated using G*Power 3.1.9.6 with an effect size of 0.15, an alpha of 0.05, 105 participants, and 4 predictors. The results indicated that the statistical power was high at 0.98. All statistical analyses were conducted with a significance level of 0.05 using SPSS 25.0 (SPSS Inc., Chicago, USA) and Amos (SPSS Statistics 25.0, SPSS Inc., Chicago, IL, USA) software packages.

## 3. Results

Table 1 presents the means and standard deviations for the measured variable in this study. There were 35 male participants (33.3%) and 70 female participants (66.7%) with an age range of 65 to 94 years and a mean age of 79.0 years. The average value of each measured variable was 3.6 for *Ikigai*, 115.2 for the PASE, 26.6 for the MMSE, 3.4 for the GDS-15, and 22.9 for the LSNS-6. 

Table 2 shows the means and standard deviations of the *Ikigai* score for the physical, psychological, and social assessments. The groups with 0 and 1 for the *Ikigai* score were excluded from the statistical analysis, as they comprised only one or two subjects, respectively.

Regarding age, gender and PASE, no characteristic trends were observed in each group. It was observed that the group with the *Ikigai* score of 2 exhibited lower values than the group with the *Ikigai* score of 3 or higher for the MMSE and LSNS-6. A trend toward lower GDS-15 values was observed as the *Ikigai* score increased above 2. The GDS-15 values of the groups with *Ikigai* scores of 4 and 5 were significantly lower than those of the group with a score of 2 (*p* = 0.045 and *p* = 0.016, respectively).

Table 3 shows the results of the correlation analysis and Spearman’s correlation coefficients (SCC) among the variables. Significant positive correlations were found between PASE and MMSE (SCC: 0.227). The LSNS-6 significantly correlated with both the MMSE (SCC: 0.325) and the GDS-15 (SCC: −0.361).

Table 4 shows the results of the multiple regression analysis after age and gender adjustment. A significant association was found only for GDS-15 (β: −0.407, *p* < 0.01). No multicollinearity was noted among the independent variables, and the variance inflation factor (VIF) values ranged between 1.282 and 1.605.

**Figure 1:**
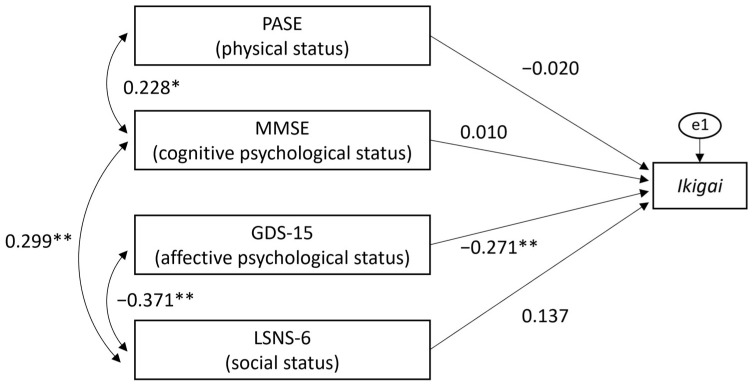
Covariance structure model examining the relationship between *Ikigai* and physical/psychological/social factors. (PASE: Physical Activity Scale for the Elderly, MMSE: Mini-Mental State Examination, GDS-15: Geriatric Depression-15 Scale, LSNS-6: Lubben Social Network Scale). Statistical significance (* *p* < 0.05, ** *p* < 0.01).

Considering the above results, a covariance structure analysis was performed to investigate a path model in which physical, psychological, and social assessments affect *Ikigai*, as shown in Figure 1. In this model, four paths from physical, psychological, and social assessments to *Ikigai* and correlations between the PASE and MMSE, the LSNS-6 and GDS-15, and the LSNS-6 and MMSE were confirmed. The path coefficients from the PASE, MMSE, GDS-15, and LSNS-6 to *Ikigai* were as follows, respectively: −0.020, 0.010, −0.271, and 0.137. The path from the GDS-15 to *Ikigai* was significant (*p* < 0.01). The adaptability of this model was good (*p* = 0.133, GFI: 0.979, AGFI: 0.895, CFI: 0.944, RMSEA: 0.091).

## 4. Discussion

*Ikigai,* in which the concept with a spiritual approach originated in Japan, has been described in various terms [11]. In this study, *Ikigai* was investigated with three aspects: physical, psychological (including affective and cognitive aspects), and social factors, in line with the definition of frailty and health, using objective assessment scales.

There have been various reports on the relationship between *Ikigai* or “having a purpose in life” and physical factors. Alimujiang reported that having a life purpose promotes healthy behaviors [34], and McKnight and Kashdan stated that it triggers one’s engagement in healthy behaviors, such as exercise, which affects one’s physical condition [35]. Boyle et al. also reported that having a life purpose can reduce the risk in the future activities and the instrumental activities of daily living [36]. The correlation between *Ikigai* and depression as an affective psychological factor has been studied in several earlier studies. Wilkes et al. reported a significant negative correlation between them [37]. Nakao et al. [38] also reported a significant negative correlation between *Ikigai* and the GDS-15, which was used in this study as a measure of affective psychological state. Therefore, *Ikigai* has been described as a factor for a better life and has been reported to have a positive impact on the maintenance of physical and affective psychological conditions.

This study indicates that affective psychological status, such as depression, has a large impact on *Ikigai* along with physical, cognitive psychological, and social conditions. Thus, it is appropriate to consider that good affective psychological status is a fundamental condition for having *Ikigai*. As García and Miralles suggested, *Ikigai* is a fundamental state—and especially a fundamental psychological condition—after which one works with the goals of “passion”, “profession”, “vocation”, and “mission”, which can positively impact one’s physical, psychological and social conditions [11]. The former is a “foundational state” and the latter are “target states” according to the opinions of Japanese researchers. The concept of “meaning of life”, which is one of the elements of *Ikigai*, is thought to include three components: cognitive, affective, and motivational [39]. The emotional component includes satisfaction, fulfillment, and happiness, all of which are listed as the mental states that comprise *Ikigai* [12]. The GDS-15, an affective psychological status assessment tool used in this study, includes questions such as “Are you basically satisfied with your life? (satisfaction)”, “Do you feel that your life is empty? (fulfillment)”, and “Do you think it is wonderful to be alive now? (happiness).” The results of this study are also understandable, as affective psychological status forms the basis for obtaining *Ikigai*.

As mentioned above, past studies have reported that a significant relationship exists between one’s physical condition, such as healthy behaviors and activities of daily living, and *Ikigai*. However, no significant relationship between PASE as a physical condition and *Ikigai* was observed. To date, there is little information available on the relationship between PASE and *Ikigai*. The average value of PASE and the age of the participants in this study were 115.2 and 79.0, respectively. In a past study with community-dwelling older adults in Japan [22], these values were 114.9 and 72.6, respectively. Although the average age in our study was slightly higher compared to the past study, the PASE was similar in these two studies. Considering these facts, regarding the relationship between PASE and *Ikigai* in our study, these results may reflect the unique characteristics in hilly and mountainous regions, where citizens support each other, interact with others, discover hobbies or life purposes, and find *Ikigai*, even if physical activity is low. As for the relationship between MMSE, which indicates cognitive function, and *Ikigai*, the results of this study showed no significant relationship between them. Several significant associations between cognitive function and *Ikigai* have been reported. Okuzono et al. [6] reported that having *Ikigai* reduced the risk of developing dementia three years later by 0.64-fold. Considering that the median MMSE value of the subjects in this study was 27 and the percentage of subjects with severe cognitive decline with the value of 20 or less was 6.7%, which were comparable to previous studies in Japan [40,41], it is possible to obtain *Ikigai* even with cognitive decline, as in the PASE described above. The results may reflect a unique characteristic of hilly and mountainous areas in Japan, where it is possible to have *Ikigai* despite cognitive decline, as in the PASE study. There is also a report [42] that found no significant relationship between psychological well-being and MMSE in patients with Alzheimer’s disease. Further research is needed to investigate the relationship between physical function, cognitive function, and *Ikigai*, including the characteristics of the older subjects. Significantly, we found that affective psychological conditions, such as depression, have large impact on *Ikigai* in this setting.

Similar to the relationship with physical factors, the relationship between *Ikigai* and social factors has been examined. Seko and Hirano reported that people who were more engaged in social activities, such as interacting with neighbors, have higher *Ikigai* [43]. In particular, older women are more likely to participate in community activities and have a higher subjective sense of health [44]. In this study, a trend toward higher LSNS-6 values was observed as the *Ikigai* score increased, but the difference was not statistically significant. Moreover, no significant association was found between the LSNS-6 results and *Ikigai* with a multiple regression analysis and covariance structure analysis. With respect to the LSNS-6’s cut-off value, a person with a score of 11 or less is considered at risk for social isolation [32]; however, based on this cut-off value, no participants in this study were at risk of social isolation. Thus, it is thought that the non-social isolation tendency of the study participants affected these results. 

Regarding the limitations of this study, the participants were a unique group of older adults living in the hilly and mountainous regions in Japan. Compared to urban and rural areas, there are many restrictions to daily life in this area, such as mobility and shopping; and the social situation does not always change throughout one’s lifetime because work life and daily life are comparatively one entity. This study did not examine the effects of confounding factors, such as vision and hearing disorders and educational background, which are thought to influence *Ikigai*, in addition to these regional characteristics. This is one of the limitations of this study and should be examined in the future. However, considering that hilly and mountainous areas account for 70% of Japan’s land area and have a high aging rate, it is important to examine *Ikigai*-related factors in older adults living in these areas. This study’s findings will be more meaningful if compared with results of future studies on older adults living in urban or rural areas. Furthermore, the differences in the number of participants in each *Ikigai* group may have resulted in a sampling bias. Although this study’s target area is a hilly and mountainous region, with limited living resources for daily life, 97.1% of the total population had moderate or high *Ikigai* (*Ikigai* score of 2–5), and only 2.9% of the participants had low *Ikigai* (*Ikigai* score of 0 or 1). Therefore, most participants in the present study had high levels of *Ikigai*; the results should be carefully interpreted.

Another limitation is regarding the assessment of *Ikigai*. In this study, physical, psychological, and social factors were assessed using the PASE, GDS-15, MMSE, and LSNS-6, which already have high validity and reliability. In this study, *Ikigai* was assessed through the self-rated score on a scale of 0 to 5. Although the question item on *Ikigai* was set based on the previous report [18], the reliability and validity of this method have not been examined. *Ikigai* is the integration of the object of *Ikigai* and the feelings associated with its object, which is difficult to assess because it has spiritual aspects in addition to elements such as the will to live, a sense of being, dreams, and a sense of fulfillment in life. Regarding the assessment of *Ikigai*, the Ikigai-9 was developed by Imai et al. for middle-aged and older adults in a specific region [45]. To date, there is no assessment that can be used globally and across generations, from adolescence to older age. As *Ikigai* is subjective, the visual analog scale may be preferable. The development of an assessment questionnaire for *Ikigai* with proven reliability and validity is needed for future studies. As mentioned above, *Ikigai* is associated with a variety of general health conditions, including mortality and physical dysfunction. In the present study, when examining *Ikigai* from a biopsychosocial perspective, an affective psychological status, such as depressive symptoms, was found to have the greatest impact on *Ikigai*. In other words, improving one’s affective psychological status as a prerequisite for achieving *Ikigai* may contribute to extending one’s healthy life expectancy. Conceivably, this study was conducted in one hilly and mountainous area, and the results were obtained from a limited area. However, this study is the first to identify the *Ikigai* of older people in such an area. Additionally, considering that hilly and mountainous areas have an extremely high aging rate, and that the aging of the population is expected to continue worldwide, we believe that the results of this study obtained from this region are highly significant.

## 5. Conclusions

**Figure 2:**
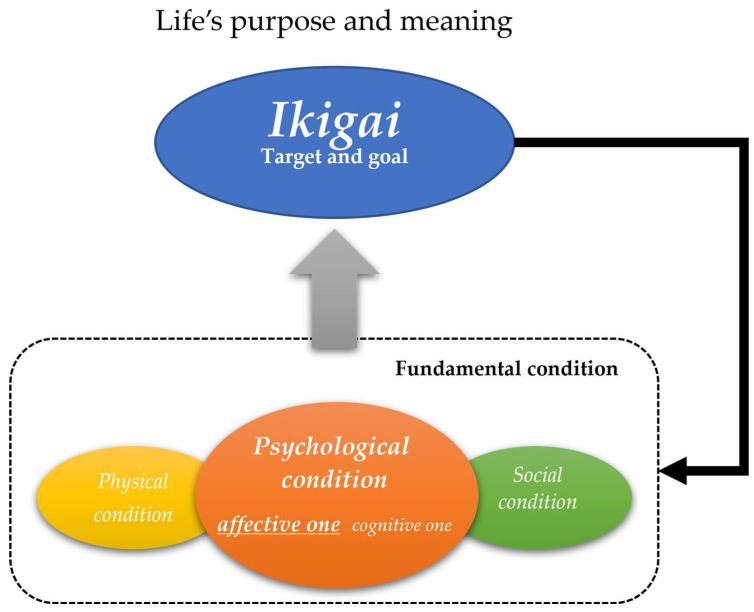
Biopsychosocial model of *Ikigai*.

*Ikigai*, which is an original Japan concept and is associated with the QoL and well-being of older adults, was analyzed through the cross-sectional study. Thus, we propose a biopsychosocial model of *Ikigai*, which indicates that depressive statuses have a large impact on *Ikigai*, along with physical, cognitive, and social conditions (Figure 2). It is appropriate to consider that an affective psychological status needs a fundamental condition for having *Ikigai*.

## Figures and Tables

**Table 1:** Participants’ characteristics.

Characteristics				
**Gender**	n		%	
Male	35		33.3	
Female	70		66.7	
**Age (years)**				
Mean ± SD	79.0 ± 6.9			
Min-Max value	65–94			
**Variable**	**Mean ± SD**	**Median**		**Min–Max**
***Ikigai***	3.6 ± 1.0	4		0–5
**PASE** **(physical status)**	115.2 ± 51.3	111.5		2.2–346.8
**MMSE** **(cognitive psychological status)**	26.6 ± 3.1	27		16–30
**GDS-15** **(affective psychological status)**	3.4 ± 2.6	3		0–11
**LSNS-6** **(social status)**	22.9 ± 4.9	24		12–30

(PASE: Physical Activity Scale for the Elderly, MMSE: Mini-Mental State Examination, GDS-15: Geriatric Depression-15 Scale, LSNS-6: Lubben Social Network Scale).

**Table 2:** Means and standard deviations of the *Ikigai* score in age, gender and four assessments: PASE, MMSE, GDS-15, and LSNS-6.

Variable	*Ikigai* Score					
	0 (*n* = 1)	1 (*n* = 2)	2 (*n* = 5)	3 (*n* = 40)	4 (*n* = 35)	5 (*n* = 22)
Age (years)	80.0	86.0 ± 2.8	79.2 ± 7.2	78.3 ± 7.6	78.7 ± 6.6	80.1 ± 6.5
Gender	M: 0, F: 1	M: 1, F: 1	M: 2, F: 3	M: 10, F: 30	M: 17, F: 18	M: 5, F: 17
PASE	144.6	111.1 ± 14.1	68.5 ± 40.5	117.1 ± 51.9	120.8 ± 58.7	112.2 ± 38.4
MMSE	25.0	21.0 ± 7.1	24.8 ± 0.8	26.7 ± 3.6	27.2 ± 2.4	26.3 ± 2.7
GDS-15	6.0	7.5 ± 5.0	6.0 ± 3.4	4.0 ± 2.8	2.9 ± 2.1 *	2.3 ± 1.6 *
LSNS-6	23.0	19.0 ± 5.7	20.6 ± 3.6	21.9 ± 5.3	24.5 ± 3.8	23.4 ± 5.4

Values in bold indicate statistical significance compared to the *Ikigai* score of 2 (* *p* < 0.05), (M: male, F: female, PASE: Physical Activity Scale for the Elderly, MMSE: Mini-Mental State Examination, GDS-15: Geriatric Depression-15 Scale, LSNS-6: Lubben Social Network Scale).

**Table 3:** Spearman’s correlation coefficients for the four assessments: PASE, MMSE, GDS-15, and LSNS-6.

Variable	PASE	MMSE	GDS-15	LSNS-6
**PASE**	1.000	0.227 *	−0.183	0.122
**MMSE**		1.000	−0.097	0.325 **
**GDS-15**			1.000	−0.361 **
**LSNS-6**				1.000

Statistical significance (* *p* < 0.05, ** *p* < 0.01), (PASE: Physical Activity Scale for the Elderly, MMSE: Mini-Mental State Examination, GDS-15: Geriatric Depression-15 Scale, LSNS-6: Lubben Social Network Scale).

**Table 4:** Summary statistics of multiple regression analysis after age and gender adjustment for *Ikigai* and the four assessments: PASE, MMSE, GDS-15, and LSNS-6.

Independent Variable	β	*p*-Value	VIF
**PASE**	0.007	0.950	1.605
**MMSE**	0.160	0.158	1.515
**GDS-15**	**−0.407**	**<0.001**	1.282
**LSNS-6**	−0.017	0.880	1.572

Values in bold indicate significant characteristics (*p* < 0.05). (β: standardized partial regression coefficient, PASE: Physical Activity Scale for the Elderly, MMSE: Mini-Mental State Examination, GDS-15: Geriatric Depression-15 Scale, LSNS-6: Lubben Social Network Scale, VIF: Variance inflation factor).

## Data Availability

The data used to support the findings of this study are available from the corresponding author upon reasonable request.
